# Extracting Symptoms of Complex Conditions From Online Discourse (Subreddit to Symptomatology): Lexicon-Based Approach

**DOI:** 10.2196/70940

**Published:** 2025-09-12

**Authors:** Bushra Hossain, Sarah M Preum, Md Fazle Rabbi, Rifat Ara, Mohammed Eunus Ali

**Affiliations:** 1 Department of Computer Science and Engineering Bangladesh University of Engineering and Technology Dhaka Bangladesh; 2 Department of Computer Science Dartmouth College Hanover, NH United States; 3 Department of Biomedical Data Science Geisel School of Medicine Hanover, NH United States; 4 Department of Computer Science and Engineering Bangladesh University of Professionals Dhaka Bangladesh; 5 Department of Obstetrics & Gynecology Bangladesh College of Physicians and Surgeons Dhaka Bangladesh; 6 Faculty of Information Technology Monash University Clayton Australia

**Keywords:** complex medical condition, polycystic ovary syndrome, online discourse, large language models, natural language processing, disease-specific symptoms, health information extraction, symptomatology

## Abstract

**Background:**

Millions of people affected with complex medical conditions with diverse symptoms often turn to online discourse to share their experiences. While some studies have explored natural language processing methods and medical information extraction tools, these typically focus on generic symptoms in clinical notes and struggle to identify patient-reported, disease-specific, subtle symptoms from online health discourse.

**Objective:**

We aimed to extract patient-reported, disease-specific symptoms shared on social media reflecting the lived experiences of thousands of affected individuals and explore the characteristics, prevalence, and occurrence patterns of the symptoms.

**Methods:**

We propose a lexicon-based symptom extraction (LSE) method to identify a diverse list of disease-specific, patient-reported symptoms. We initially used a large language model to accelerate the extraction of symptom-related key phrases that formed the lexicon. We evaluated the effectiveness of lexicon extraction against human annotation using a Jaccard index score. We then leveraged BERT-Base, BioBERT, and Phrase-BERT–based embeddings to learn representations of these symptom-related key phrases and cluster similar symptoms using k-means and hierarchical density-based spatial clustering of applications with noise (HDBSCAN). Among the different options explored in our experiments, BioBERT-based k-means clustering was found to be the most effective. Finally, we applied symptom normalization to eliminate duplicate and redundant entries in the comprehensive symptom list.

**Results:**

In a real-world polycystic ovary syndrome (PCOS) subreddit dataset, we found that LSE significantly outperformed state-of-the-art baselines, achieving at least 41% and 20% higher *F*_1_-scores (mean 86.10) than automatic medical extraction tools and large language models, respectively. Notably, the comprehensive list of 64 PCOS symptoms generated via LSE ensured extensive coverage of symptoms reported in 7 reputable eHealth forums. Analyzing PCOS symptomatology revealed 28 potentially emerging symptoms and 8 self-reported comorbidities co-occurring with PCOS.

**Conclusions:**

The comprehensive patient-reported, disease-specific symptom list can help patients and health practitioners resolve uncertainties surrounding the disease, eliminating the variability of PCOS symptoms prevailing in the community. Analyzing PCOS symptomatology across varied dimensions provides valuable insights for public health research.

## Introduction

### Background

The rise of health 2.0 has significantly increased the consumption of health-related information on social media in recent years. Worldwide, 34.7% of adult internet users frequently search for health-related information online, with approximately 58.5% of adults in the United States specifically engaging in such searches as of 2023 [1,2]. Analyzing online discourse on health-related topics offers valuable insights into the lived experiences and perspectives of affected individuals [3-5]. The critical findings of these analyses are particularly significant for understanding complex medical conditions, such as different types of cancer, cognitive and mental health conditions, autoimmune disorders, and metabolic and endocrinological conditions. These medical conditions often manifest with diverse symptoms that vary significantly among patients [6-8]. Furthermore, there is often no consensus among established medical sources regarding the full range of possible symptoms for such conditions [9,10]. This variability poses challenges for patients, health care providers, and researchers seeking a comprehensive understanding of these symptoms and their characteristics. Thus, the study and analysis of symptoms associated with a disease, referred to as *symptomatology*, is crucial for understanding complex diseases [11]. We argue that a robust approach to extracting disease-specific symptoms from individuals’ lived experiences shared on social media can address uncertainties surrounding complex medical conditions. This approach would also allow for the identification of symptoms that patients associate with a condition (eg, brain fog with post–COVID-19 condition), prevalence of symptom co-occurrence (eg, unexplained weight loss and pain with cancer), and how symptoms vary based on patients’ comorbidities and preexisting conditions.

Recent advancements in natural language processing (NLP) have enabled public health researchers to explore medical information extraction tools [12-15] and develop novel techniques [16-18] for analyzing online health discourse, including patient-reported data from social media. However, off-the-shelf medical information extraction tools primarily excel in extracting well-known medical entities (eg, diseases, drugs, and basic symptoms). Their limitations become evident with granular, patient-centric details (eg, unusual symptom descriptions, implicit context, or subtle adverse events), which often require specialized rules or retrained models for accurate detection [19-22]. Modern transformer-based models used with medical named entity recognition have shown promising results in symptom extraction [23,24]. However, these models typically require large amounts of domain-specific data and are mostly trained on clinical notes (electronic health records), which limits their effectiveness when dealing with colloquial health discourse generated by patients [7,23-25]. A family of bidirectional encoder representations from transformers (BERT)–based models fine-tuned on colloquial data markedly improves symptom recognition in informal texts; nevertheless, their error analysis indicates that the best-trained models still capture only approximately 25% to 50% of lengthy, figurative symptom phrases and continue to misclassify many subtle or previously unseen expressions (eg, “mild difficulty taking deep breaths” or “chest again felt a little tight”) [26]. A recent NLP pipeline [27] used to extract symptoms from patient narratives on social media proposed a dual-corpus symptom lexicon containing keywords (eg, *fever* and *cough*) and related modifiers (eg, *mild* or *severe*) followed by applying a rule-based postprocessing step to merge the 2. However, this fixed rule-based approach may struggle to report symptoms whenever people describe them implicitly, with spelling noise, or in new phrasing that never entered the seed lists (eg, “can’t breathe properly” or “my lungs felt on fire”).

While the existing lexicon-based approaches have successfully used predefined resources and deep learning frameworks for broad public health surveillance, their reliance on explicit wording limits their ability to capture the full range of informal expressions. On the other hand, large language models (LLMs) have significantly accelerated medical NLP research, especially in the field of information extraction [16,17,21,28]. While some studies have demonstrated the potential of LLMs for health information extraction from social media [29,30], they face challenges when extracting specific contexts such as health advice and disease-specific treatment [12,16].

### Objectives

To overcome the aforementioned limitations of symptom extraction from social media discourse, we propose a novel NLP pipeline that systematically combines the capabilities of LLMs and other NLP methods with feedback from domain experts, resulting in a scalable and practical solution to the problem of symptom extraction from patient-reported social media discourse.

While the proposed pipeline can be applied to explore a plethora of medical conditions for which there is an abundance of patient-reported online discourse, we focused on a common chronic condition with significant uncertainty, namely, polycystic ovary syndrome (PCOS), as a use case in this paper. According to the World Health Organization [31], an estimated 116 million women (3.4% of reproductive-age women) worldwide are affected by PCOS, and its prevalence is on the rise. Despite its widespread impact, the full spectrum of PCOS remains insufficiently explored [32]. Patients report a diverse range of symptoms that vary in severity depending on previous medical history and their effect on daily life. The combination of varied symptoms, insufficient treatment guidelines, and instances of medical gaslighting often leads to psychological distress, prompting many individuals to seek support and share experiences on social media platforms [33], including Reddit. Existing research leveraging social media data to study PCOS is still underdeveloped [4,34-36]. This creates a unique opportunity to systematically identify self-reported symptoms and related symptomatology based on the lived experiences of thousands of individuals and, thereby, inform the clinical understanding of this complex medical condition. We make 3 primary contributions. First, we developed a novel lexicon-based symptom extraction (LSE) method to identify diverse disease-specific symptoms from individuals’ experiences shared on social media. This method can effectively detect even subtle symptoms mentioned in user discourse. We applied our LSE method to a novel PCOS subreddit dataset, achieving statistically significant improvements over state-of-the-art medical NLP baselines.

Second, we conducted an in-depth analysis of peer discussion threads on the extracted PCOS symptoms to reveal a set of lesser-known yet prevalent symptoms that individuals associate with PCOS and associated co-occurring symptoms and comorbidities. These findings can contribute to clinical research and public health research in a community-informed way.

Third, we curated a novel Reddit dataset containing 59,962 and 641,441 relevant posts and comments, respectively. This dataset captures self-reported PCOS symptoms and their context from 68,010 affected individuals. In addition, we curated a human-annotated, labeled dataset of 1000 posts of patient-reported symptoms related to PCOS based on guidance from domain experts and patient input. We release these resources in a public domain. These unique, rich resources can enable medical NLP and computational health researchers to further investigate the symptomatology of complex medical conditions identified and analyzed online.

## Methods

### Data Collection and Preprocessing

We selected Reddit as a source of data because of its anonymous nature. It encourages users to share personal experiences on stigmatized topics [4,37]. The data collection process began with the identification of the r/PCOS subreddit by considering the group posts’ availability and popularity among 153,000 members engaging in discussions on PCOS. We systematically collected data from this subreddit using the *Python Reddit API Wrapper* package (Python Software Foundation) and the Pushshift application programming interface (API) [38,39]. We obtained all the posts and comments from the r/PCOS subreddit from August 4, 2010, to December 31, 2022. This timeline was chosen to ensure a comprehensive capture of recent as well as previous discussions. For each post, we retained the date, flair name, unique post ID, post title, post content, score, upvotes, and comments due to their utility and relevance.

During preprocessing, we discarded entries with deleted posts or those in which the post content had <5 words as the brevity poses challenges to deriving meaningful insights from posts. We also removed posts containing irrelevant text (eg, polls, survey links only, or URLs). The URL-heavy posts were classified using a rule-based heuristic combining personal pronoun frequency and URL-to-text ratio. We provide a summary of the steps to remove irrelevant text from social media data in Table S1 in Multimedia Appendix 1. This filtering process enabled us to focus on patient-authored, experience-rich discussions. Finally, we obtained a primary dataset comprising 59,962 posts and 641,441 comments by 68,010 active unique users. Identifying task-relevant data is crucial for data-driven approaches [12] as it significantly impacts the efficiency of downstream tasks. We leveraged Reddit’s post “flair” option to filter relevant data. On Reddit, a post flair is a customizable text or icon that appears next to a post title to provide additional context or categorization, similar to hashtags available on other platforms. We reviewed 30 randomly selected posts from several flairs of the PCOS subreddit. We identified that, among all the flairs, posts in the “General/Advice” flair were more likely to share knowledge and seek advice on symptoms. Further investigation on the most frequent unigrams of these flairs supported our qualitative analysis, where the presence of symptom-related unigrams such as “period,” “symptom,” “doctor,” “birth,” “control,” and “diagnosed” was predominantly observed in the “General/Advice” flair. The distribution of frequent unigrams in the “General/Advice” flair is shown in Figure S1 in Multimedia Appendix 1. On the basis of these findings, we chose the “General/Advice” flair for extracting a wide range of symptoms. In addition, the “General/Advice” flair is more popular in the community as it contains the highest number of posts, as shown in Table S2 in Multimedia Appendix 1. This selection ensured a broad range of symptoms and valuable insights. Finally, we developed a corpus of 10,500 posts for the symptom identification task, randomly selected from the posts with the “General/Advice” flair.

### Our Method: LSE

**Figure 1:**
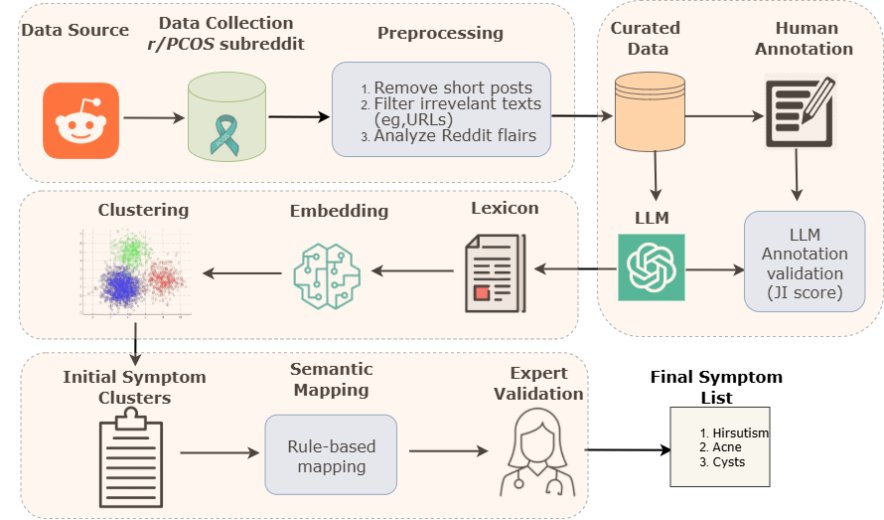
Steps of the lexicon-based symptom extraction method. JI: Jaccard index; LLM: large language model.

We propose an LSE method to analyze self-reported symptoms of a complex medical condition using social media discourse. Our proposed method consists of three main steps: (1) symptom-related lexicon extraction, (2) symptom identification, and (3) symptom normalization, as illustrated in Figure 1.

#### Symptom-Related Lexicon Extraction

We leveraged symptom-related key phrases for lexicon extraction as it helped distill the most relevant terms and phrases associated with physical manifestations from user narratives. We focused on extracting the key symptom-related phrases instead of the symptoms as the latter results in extracting (1) phrases that are not relevant and (2) repetitive mentions of the same symptoms, rendering the subsequent analysis challenging. Compared to the tedious and time-intensive task of manual extraction, LLMs emerge as a more economical choice, offering consistency in their responses, especially when the temperature setting is near 0 [40,41]. We used the OpenAI API *gpt-3.5-turbo-16k* [42] to annotate 10,500 user posts. To ensure precision in identifying the physical manifestation of the condition, we emphasized physical symptoms while excluding psychological aspects in our base prompt using a zero-shot prompting strategy (Figure S2 in Multimedia Appendix 1). The API responses were stored in a JSON line format containing key phrases and corresponding posts. This process yielded a lexicon of 30,370 symptom-related key phrases that we used in the subsequent steps.

We resorted to human annotation to validate the extraction conducted by ChatGPT (OpenAI). We employed 2 graduate students in the science, technology, engineering, and mathematics field particularly active in women’s health–related social media forums. Before annotation, they underwent multiple training sessions led by a domain expert specializing in computational health. These sessions were designed to refine their understanding on key phrase extraction and promote annotation consistency. On a sample set of 30 posts, the interannotator agreement reached 92% in a relaxed match. Finally, we randomly selected 500 entries from 10,500 user posts. We recognize that many social media and health NLP studies use larger annotated sets, often containing 2000 to 4000 messages, and few drop below 1000 annotations [12,43-45]. However, those datasets are typically used to validate end-to-end model performance, whereas our 500-post sample supported a specific component of our pipeline. We deliberately divided tasks between the LLM and human experts to balance coverage and precision while remaining practical as (1) the cost in terms of time and resources was significant (eg, annotating just 500 entries required 4 training sessions × 3 hours = 12 hours, 35 hours for literature study and documentation, and 208.3 hours approximately for annotation per post; 500 posts × 25 minutes), making larger samples prohibitively expensive; (2) we observed a significant drop in new symptom-related key phrase discoveries after the first 200 posts, mirroring the classic “diminishing returns” shown in Table S3 in Multimedia Appendix 1, also evident in social media symptom mining and related tasks [46]; and (3) during manual inspection, we observed minimal disagreement as the annotation task was primarily syntactic rather than interpretive. Taking into consideration the above 3 points, we assigned 250 unique posts to each annotator to minimize bias and enhance the quality of our annotated set.

We choose the Jaccard index (JI) to measure the similarity between key phrases extracted by ChatGPT and the human annotators [12]. This required normalizing the extracted key phrases (eg, changing “pressure in pelvic area” to “pelvic pressure”) to maintain homogeneity in both lists. We computed the JI similarity for each post in the sample using exact key phrase match. Table 1 shows 3 posts from our sample set with extracted key phrases and their corresponding JI scores. The average JI score was 74%, indicating moderate lexical similarity between the key phrases extracted by ChatGPT and the human annotators considering the complexity of the task [47]. Our manual investigation of all missed or incorrectly extracted key phrases by the LLM revealed 5 distinct error categories, as outlined in Table S4 in Multimedia Appendix 1. For instance, in row 1 of Table 1, the LLM missed “absence of period” as it was implicitly mentioned in the post as “haven’t had a period since December.” Similarly, in row 3, “insulin resistance” is an ambiguous expression to be accepted as a symptom. However, a deeper analysis revealed 3 findings supporting the acceptability of the lexicon.

First, post-level JI analysis; our manual investigation showed that 12.6% (63/500) of the posts had a JI score of 0, which substantially affected the average JI. We found that most of these JIs with a value of 0 resulted from very short posts (eg, “Does anyone know if weightloss in PCOS helps or throws off hormones?”) and were annotated as *no symptom* by humans, whereas the LLM incorrectly extracted the key phrases *weightloss* and *hormones*. The median JI score across all posts was 1.0 (IQR 0.5-1.0), indicating strong agreement between the LLM and human annotations in most cases.

Second, lexicon-level robustness; we observed that the LLM missed a key phrase in a post, resulting in a mismatch, but extracted the same key phrase in another post. Similar cases were found for human annotation, resulting in incorrect extraction for the LLM. We identified such cases for 61% (104/172) of the total missed or incorrectly extracted key phrases, which affected the average JI. As the final lexicon aggregates key phrases across all posts, these disagreements at the post level did not propagate to the lexicon level. When comparing the LLM-derived lexicon with the human-annotated lexicon, we observed a JI of 0.89, demonstrating strong alignment between the 2. This high agreement confirmed that discrepancies in per-post extraction had minimal impact on the final lexicon’s accuracy and coverage.

Third, downstream impact on symptom list; the frequency of unique incorrectly extracted key phrases in the lexicon was very low (maximum of 3). Consequently, their influence on the cluster centroids was negligible. Because our symptom list was derived by selecting the most frequent phrases within each cluster, the effect of noisy or rare phrases was minimized. Such key phrases either formed microclusters (eg, *ultrasound*) that were discarded during the normalization step or were absorbed into broader, validated symptom cluster categories.

**Table 1:** Sample excerpts of human and ChatGPT annotations of posts. The Jaccard index (JI) was calculated for the similarity between the 2 annotations based on the extracted key phrases.

Post	Human annotation	ChatGPT annotation	JI score
“I haven’t had a period since December, however in the past week I keep getting really bad cramping with sharp pains (almost feels like my ovaries), but no period...has anyone else experienced this? Is this normal?”	“Absence of period,” “cramping,” and “sharp pains”	“Cramping” and “sharp pains”	0.67
“I’m 22, F and over the years, I have gained 50 pounds. Recently, my acne is getting worse and I feel pressure in my pelvic area. I am trying to lose weight and thereby started working out but the scale is still not budging.”	“Weight gain,” “acne,” and “pelvic pressure”	“Weight gain,” “acne,” and “pelvic pressure”	1.0
“Due to PCOS and insulin resistance, Does anyone else get symptoms like they’re getting their period (sore breasts, crampy feeling) and just overall malaise when you’re ‘due’ for your period you don’t actually get?”	“Insulin resistance,” “sore breasts,” and “crampy feeling”	“Sore breasts,” “crampy feeling,” and “malaise”	0.5

In summary, while there were post-level discrepancies, the high lexicon-level JI (0.89) and the robustness of frequency-based clustering ensured that the final symptom list remained accurate and representative of human annotation without prohibitive annotation burden.

#### Symptom Identification

##### Overview

In this step, we identified a distinct set of disease symptoms using the symptom-related lexicon. As the key phrases in the lexicon were captured exactly as mentioned in users’ posts, many were semantically similar with different phrasing. For example, key phrases such as *cystic ovary*, *ovarian cysts*, and *multiple cysts in ovaries* have the same semantic meaning with different surface forms. Thus, we used clustering to categorize the semantically similar key phrases and identify such categories as unique symptoms. To ensure accurate clustering, it is crucial to generate meaningful representations of symptom-related key phrases that effectively capture their semantic meaning. To achieve this, we used embeddings, which convert words into numeric representations in lower dimensions that preserve their contexts. The process of embedding and clustering the key phrases is described in the following sections.

##### Choosing the Right Embedding

**Figure 2:**
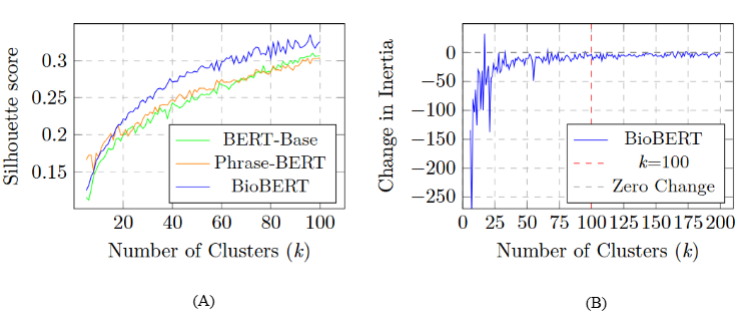
Characteristic analysis of embedding methods for selecting optimal clustering methods—(A) silhouette score analysis of different embeddings on k-means and (B) rate of change in inertia (within-cluster sum of the squares) decreasing as the number of clusters increases. BERT: bidirectional encoder representations from transformers.

We explored and evaluated widely used transformer models such as BERT-Base [48], Phrase-BERT [49], and BioBERT [50] to generate embeddings of symptom-related key phrases. We observed the performance of these embeddings by applying various clustering methods. For k-means clustering, we analyzed the silhouette score for each BERT-based embedding. The silhouette score considers both within-cluster and intercluster variances to determine cluster quality [51]. We plotted silhouette scores of different embedding approaches, shown in Figure 2A. The curves for BERT-Base and Phrase-BERT show a general increase but noticeable fluctuation, representing inconsistency in the results. In contrast, BioBERT shows a consistent and uniform ascent compared to the other 2 in the silhouette curve. Furthermore, applying L2 normalization during embedding improved the uniqueness of the clusters [48]. The consistent superiority of BioBERT across all clustering approaches steered our decision to adopt BioBERT as our embedding method.

##### Selecting the Optimal Clustering Method

**Figure 3:**
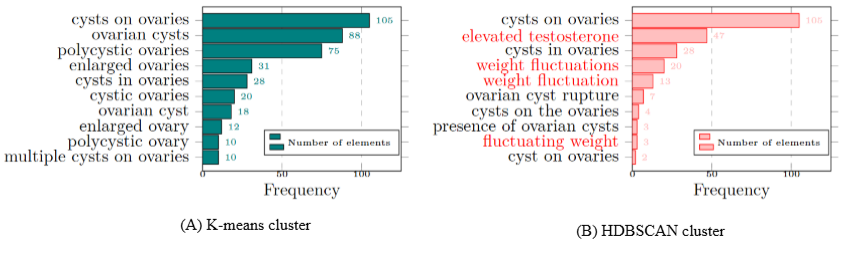
Comparison of elements within the cluster "cysts on ovaries" between 2 clustering methods: (A) k-means, containing semantically similar key phrases that are evenly distributed, and (B) hierarchical density-based spatial clustering of applications with noise, containing key phrases conflicting with the cluster name, highlighted in red, and showing more uneven distribution.

We exploited k-means and hierarchical density-based spatial clustering of applications with noise (HDBSCAN) with BioBERT embeddings for clustering symptom-related key phrases. Comparing k-means and HDBSCAN is fairly tricky as their hyperparameters (number of clusters vs density thresholds) are fundamentally different. Although it is widely known that HDBSCAN performs better for noisy social media data as it filters out “low-density” points as noise, the choice of clustering method depends on the downstream task and the performance on specific data. In Table S5 in Multimedia Appendix 1, we report the performance of HDBSCAN under a grid of *min_cluster_size* and *min_samples* settings with the observations of cluster numbers, density-based clustering validation (DBCV) score, and noise. The DBCV score measures how dense and well separated core clusters are in a density-based clustering method [52]. Thus, HDBSCAN with lower *minimum_cluster_size* increases in DBCV scores indicates compactness of the clusters but may generate spurious microclusters. Manual observation shows that HDBSCAN with lower *minimum_cluster_size* provides better intracluster coherence but reduce intercluster separation and generate more semantically similar clusters, such as *increased testosterone* and *elevated level of testosterone*. In contrast, an increasing *minimum_cluster_size* results in lower DBCV scores. As a result, the number of clusters decreases, which may contribute to losing meaningful clusters by being too strict on cluster size. For such instances, elements of the *cysts on ovaries* cluster represents less coherence and semantic similarity in HDBSCAN compared to k-means shown in (Figure 3). In our use case, HDBSCAN labeled 31% to 56% of the points as noise across settings. This affected the downstream task as our goal was to maximize symptom coverage. On the other hand, k-means has no noise filtering, which ensures maximum coverage of symptoms. In addition, we implemented k-means using the cosine distance to improve the cluster uniqueness with fewer duplicates compared to using the Euclidean distance [53].

##### Sensitivity and Stability Analysis in Choosing the *k* Value

We varied the number of clusters (*k*) to determine the optimal number based on the silhouette score (in Figure 2A) and rate of change in inertia (in Figure 2B). In our PCOS use case, we identified that, after *k*=100, the change in inertia became flat and the rate of increment in silhouette score was also negligible. Therefore, we set *k* to range from 10 to 100, and at *k*=96, we observed an optimal silhouette score. In addition, we explored a –10 to +10 window for *k*=96 to verify the sensitivity of the resulting clusters. Across *k*=86 to 106, the silhouette score remained within approximately –0.01 to +0.01 of its value at *k*=96, and the inertia curve showed minimal change, confirming that the choice of *k* sat in the middle of a stable region. In addition, we report the bootstrap stability results for 3 representative choices between *k*=86 and 96. All 3 settings yielded high median Adjusted Rand Index (ARI) values (≥0.75) and narrow 95% CIs, demonstrating that cluster assignments are largely consistent when up to 20% of the data are held out. We provide a summary of the sensitivity and stability analysis of the choice of *k* in k-means in Table S6 in Multimedia Appendix 1.

#### Symptom Normalization

##### Overview

Each of the obtained clusters represented a symptom as the elements of the clusters were semantically similar. We considered the most frequent element of the cluster as the key symptom. The justification for selecting the most frequent element as the key symptom was 2-fold. First, its occurrences were significantly higher than those of other elements within the cluster. For instance, occurrences of *cysts on ovaries* compared to other elements within the cluster in Figure 3A were negligible. Second, all elements within a cluster consistently pointed toward the same physical condition as the key symptom. In the k-means cluster in Figure 3A, all the cluster elements referred to ovarian cysts. However, there were repetitions of symptoms in the obtained list. Therefore, we conducted symptom normalization by applying semantic mapping and validation by experts, as outlined in the following sections.

##### Applying Semantic Mapping

We applied semantic mapping of clusters, a manual process that involves merging symptoms with similar meanings and concepts using Unified Medical Language System Metathesaurus synonyms to avoid repetition. Through progressive discussions with our medical specialist, we formalized 4 semantic mapping rules suitable for this task. For instance, *irregular bleeding*, *irregular menstrual cycles*, and *irregular cycles* were mapped to *irregular periods* using rule 1, “synonym merging.” Applying these rules, we consolidated 11 such mappings from the initial 96 PCOS symptoms to 68 unique symptoms. The complete rule set is provided in Table S7 in Multimedia Appendix 1 to ensure reproducibility.

##### Validation by an Expert

We further refined the symptom list by validating the relevance of the lived experiences of patients to the disease. One of our coauthors, a gynecologist with >10 years of experience in treating PCOS, conducted this validation step. The expert’s role was to discard symptoms that were not related to the disease and resolve ambiguities between similar symptoms. For instance, the medical specialist discarded 4 irrelevant symptoms—*ultrasound*, *birth control*, *low-carb diet*, and *neck*—by manually investigating the cluster elements, resulting in a comprehensive list of 64 unique PCOS symptoms.

### Ethical Considerations

We acknowledge that the research on symptom extraction from social media has multifaceted ethical concerns and requires careful attention to privacy, consent, and the responsible use of data and technology. The research was approved by Bangladesh University of Engineering and Technology's Institutional Review Board. Although Reddit is an anonymous platform, we ensured that no personally identifiable information was inadvertently revealed during data collection, analysis, or publication. All Reddit posts and comments cited as examples in this paper were paraphrased to protect user privacy. We also recognize that the demographic composition of Reddit users might not be fully representative of the broader population with PCOS, potentially limiting generalizability. Therefore, findings derived from Reddit discourse should be generalized cautiously to other populations or settings. In addition, we recognize that any biases in the dataset and the proposed model are unintentional. As the outcomes of this work are based on social media content, we emphasize the superiority of medical evidence–based facts in cases of conflict, particularly concerning direct clinical practice and medical diagnosis. We strongly encourage the use of the model for research and exploratory purposes, serving as a foundation for further developments in computational health research.

## Results

### Test Data Curation and Ground Truth Annotation

We initially separated 1000 sample posts of various lengths from our dataset for results evaluation. Considering the influence of post length on symptom identification, we categorized the posts into three groups: (1) short posts of <100 words, (2) medium-length posts of between 100 and 300 words, and (3) long posts of >300 words. Post length distribution in the dataset revealed that 45% (450/1000) of the posts belonged to the medium-length category (Table S8 in Multimedia Appendix 1). To ensure a balance across all categories, we selected 500 posts from the medium-length category and 250 posts from each of the short and long post categories for curating test data.

We recruited a medical graduate with domain knowledge and a patient with PCOS lived experience to perform annotation. Before annotation, both annotators participated in progressive training sessions and interactive discussions with medical specialists to refine their understanding and improve annotation consistency. During this phase, the medical specialist primarily identified 3 potential disagreements between the annotators. These observed disagreements were instances in which (1) the medical graduate often used clinical terminology (eg, “amenorrhea”) whereas the patient used informal language (eg, “no period for months”); (2) the patient included self-reported experiences (eg, “hypothyroidism”) that lacked clinical validation whereas the clinician strictly adhered to established PCOS diagnostic criteria; and (3) the medical graduate inferred implicit symptoms (eg, laboratory test results) from domain knowledge, which was lacking in the patient annotation. Given our goal to prioritize the capture of patient-experienced symptoms, medical specialists suggested resolving these discrepancies by (1) avoiding overly technical clinical terms unless they were explicitly mentioned, (2) retaining self-reported experiences unless they were clinically invalid, and (3) including implicit symptoms as long as they were related to PCOS. Following these guidelines, both annotators extracted symptoms independently. We adopted the combination of both annotators’ symptom lists per post to maximize coverage and ensure the consistency and reliability of the ground truth dataset.

In our ground truth annotation, annotators were not restricted to predefined symptom lists. They identified any symptoms that they perceived as PCOS related, resulting in free-text labels. Moreover, each post included multiple symptoms, making the traditional Cohen κ not suitable. Instead, we evaluated interannotator agreement using relaxed-match *F*_1_-score due to the generative nature of our annotation [16,54,55]. We used BioBERT-based semantic similarity with the medical graduate’s annotation as a reference. We achieved an *F*_1_-score of 0.82, with a recall of 0.84 at a similarity threshold of 0.6, indicating substantial agreement considering the realistic complexity inherent in patient-derived, generative-nature symptom annotation.

### Symptom Comparison: LSE With eHealth Forums

LSE resulted in a comprehensive list of 64 unique PCOS symptoms (Table 2) from 10,500 user posts. We also determined the unique users reporting each symptom in our primary dataset, with counts ranging from 31 to 16,001. This confirmed that LSE captures widely experienced symptoms, whereas less common symptoms are not ignored. In addition, this list provides a wide range of coverage as we cross-checked it with 7 evidence-based consumer health websites: the World Health Organization [31]; the Mayo Clinic [56]; WebMD [57]; the *International Classification of Diseases, 10th Revision* [58]; Johns Hopkins Medicine [59]; the National Health Service [60]; and the Centers for Disease Control and Prevention [61]. All symptoms mentioned in these sources were included in our list except for 4: sleep apnea, high blood pressure, low progesterone, and dandruff. Upon examining the clusters, we found that these 4 symptoms were represented within their respective key clusters—*insomnia*, *high cholesterol*, *low estrogen*, and *high DHEA*—due to their close medical relevance. Furthermore, all the forums recognized the 10 most frequently reported symptoms in our dataset (Table S9 in Multimedia Appendix 1). On the other hand, we identified 28 symptoms not acknowledged by any of the eHealth forums that we mention as potentially emerging symptoms in the Discussion section. Thus, our comprehensive symptom list reflects the diversity of self-reported PCOS lived experience symptoms and aligns closely with authoritative medical sources, helping standardize the variability in the symptom descriptions.

**Table 2:** Comprehensive list of 64 polycystic ovary syndrome symptoms extracted via lexicon-based symptom extraction. The counts represent the number of unique users reporting each symptom across the primary dataset. The symptoms in italics represent potentially emerging symptoms.

Symptom	Unique users reporting the symptom, n
Hirsutism	16,001
Acne	14,256
Cysts on ovaries	13,781
Pain	13,495
Weight gain	13,150
Mood swings	11,523
Insulin resistance	9859
Irregular period	9702
Infertility	7558
Cramps	7043
Diabetes	6690
Spotting	6469
*Hypothyroidism*	5366
No period	4846
Anovulation	4755
*Nausea*	4600
*Cravings*	4566
Fatigue	4342
Bloating	4048
Headaches	4028
Heavy period	3846
Hair loss	3517
High testosterone	2794
Skin issues	2695
Hormone imbalance	2648
Inflammation	2457
*Endometriosis*	2303
*High DHEA* ^a^	2231
*Loss of appetite*	2013
*Painful periods*	1906
Insomnia	1513
*Dizziness*	1471
*Night sweats*	1457
*Stomach pain*	1374
*Blood clots*	1187
*Anemia*	1162
*Brain fog*	1158
*Vomiting*	1134
High androgen	1121
Oily skin	912
*Back pain*	843
*Digestive issues*	826
*Constipation*	813
Pelvic pain	742
Belly fat	731
Acanthosis nigricans	616
High cholesterol	613
High insulin	575
*Low libido*	575
*Abdominal pain*	564
*Ovary pain*	496
*Dry skin*	480
High estrogen	480
*Stretch marks*	439
Low estrogen	436
Brown discharge	426
Skin tags	399
*Breast tenderness*	376
*High prolactin*	331
*Pain during sex*	261
*Frequent urination*	160
*Redness*	108
*Weight loss*	45
High LH^b^ level	31

^a^DHEA: dehydroepiandrosterone.

^b^LH: luteinizing hormone.

### Baseline Comparisons

#### Overview

We compared the performance of LSE with leading baselines, including 2 popular off-the-shelf models—Amazon Comprehend Medical (ACM) [62] and Google Healthcare Natural Language API (GHNLP) [63]—in their default configuration. In addition, we evaluated both open-source and closed-source LLMs, namely, Llama 3.1 (8B) and GPT-4o in a zero-shot, near-0 temperature (0.25) setting. We identified symptoms in each post of the test dataset using all the baselines for comparison with the ground truth. For LSE, we used GPT-4o to identify symptoms guided by the comprehensive symptom list obtained through our method. Therefore, it was not strictly zero-shot but, rather, a form of lexicon-guided prompting. The model’s task was to match symptoms in the text against the LSE-provided symptom lexicon. To ensure reproducibility, we included the LLM prompt templates used for the baseline comparisons (GPT-4o and Llama 3.1), shown in Figure S3 in Multimedia Appendix 1, and LSE evaluation using GPT-4o, as detailed in Figure S4 in Multimedia Appendix 1. We evaluated competitive models using standard performance metrics—precision, recall, and *F*_1_-score. To measure the similarity between symptoms, we used BioBERT-based semantic similarity as it performs optimally with medical data. Given the nuanced nature of symptom expressions, we set the different semantic similarity thresholds at 0.4, 0.6, and 0.8 for match acceptance, and the corresponding performance results are reported in Table 3. Experimental results show that LSE outperformed all baselines significantly across all metrics at every threshold level. As expected, increasing the similarity threshold imposed stricter matching criteria, resulting in fewer accepted symptom matches. Consequently, the performance of all baseline models dropped significantly with higher thresholds. In contrast, the performance of LSE decreased only marginally, highlighting the stability and robustness of our framework. For all subsequent analyses in this study, we adopted a semantic similarity threshold of 0.6 as the default setting. Several additional useful insights from the results are discussed in the following sections.

**Table 3:** Comparing lexicon-based symptom extraction (LSE) with off-the-shelf medical information extraction tools and large language models in terms of precision, recall, and F1-score across different post lengths.

Model		Post length									
		≤100 words			>100 to 300 words			>300 words			For all length, mean *F*_1_-score
		Precision	Recall	*F*_1_-score	Precision	Recall	*F*_1_-score	Precision	Recall	*F*_1_-score	
**Similarity threshold of 0.4**											
	ACM^a^	55.27	79.85	61.21^b^	39.28	89.50	51.30	31.77	91.21	45.36	52.30
	GHNLP^c^	59.96	63.41	58.69^b^	49.39	65.79	51.76	45.29	68.30	51.25	53.37
	Llama 3.1 (8B)	53.26	73.00	57.38	62.49	79.11	65.91^b^	63.07	74.00	64.66	63.47
	GPT-4o	64.15	60.25	60.77	79.61	74.72	74.81	84.21	71.62	75.33^b^	71.41
	LSE	86.09	85.02	84.60	87.85	88.54	86.89	92.09	90.76	90.33^b^	87.17^d^
**Similarity threshold of 0.6**											
	ACM	48.63	68.61	53.45^b^	33.47	76.29	43.85	28.85	81.14	40.30	45.37
	GHNLP	47.82	49.54	46.64^b^	35.52	46.78	37.31	35.24	53.00	40.02	40.32
	Llama 3.1 (8B)	48.20	63.42	51.60	56.47	71.48	59.64^b^	55.86	64.96	57.27	57.04
	GPT-4o	59.05	55.63	56.02	72.11	68.86	68.57	77.97	66.86	70.14^b^	65.80
	LSE	84.89	83.82	83.40	86.64	87.42	85.78	91.20	89.57	89.42^b^	86.10^d^
**Similarity threshold of 0.8**											
	ACM	37.66	51.10	41.13^b^	20.68	46.07	26.87	18.58	52.81	26.63	30.38
	GHNLP	37.85	40.01	37.62^b^	24.45	33.15	26.18	25.83	39.23	29.54	29.88
	Llama 3.1 (8B)	41.12	52.23	43.60^b^	40.81	51.59	43.13	40.39	45.41	40.96	42.71
	GPT-4o	49.76	47.88	43.60	55.17	52.12	52.21	60.49	51.07	54.18^b^	51.60
	LSE	80.00	79.33	78.81	83.04	83.29	82.02	86.78	85.33	85.12^b^	81.99^d^

^a^ACM: Amazon Comprehend Medical.

^b^For these values, the highest value obtained by a model among the 3-length range.

^c^GHNLP: Google Healthcare Natural Language Application Programming Interface.

^d^For these values, the LSE obtained the highest *F*_1_-scores among all models in all the threshold-settings.

#### Off-the-Shelf Models Tend to Overpredict

**Figure 4:**
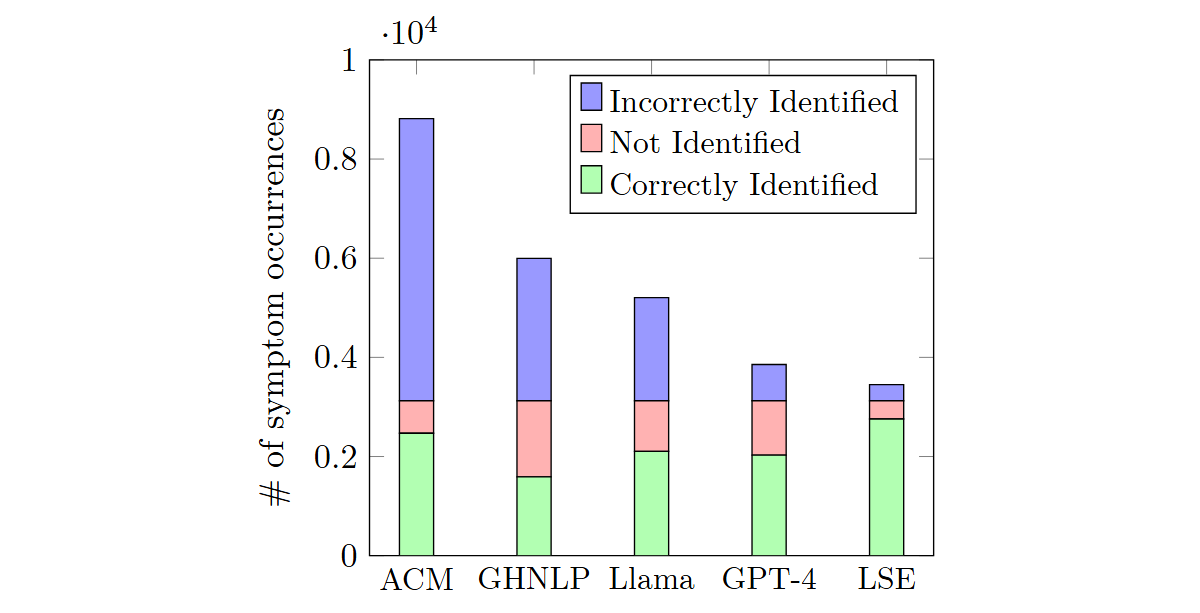
Performance comparison of different models in identifying symptom occurrences from 1000 sample posts. Each bar represents the number of correctly identified, not identified, and incorrectly identified symptoms stacked on top of each other. ACM: Amazon Comprehend Medical; GHNLP: Google Healthcare Natural Language Application Programming Interface; LSE: lexicon-based symptom extraction.

The GHNLP and ACM models largely extracted generic symptoms that were not disease specific, such as *pregnancy*, *cold*, *sick*, *illness*, and *discomfort*. In addition, they extracted mental conditions such as *anxiety*, *overwhelmed*, and *feeling bad* as PCOS symptoms. As a result, they identified more symptoms (4.84 and 9.05 per post by GHNLP and ACM on average, respectively) compared to the ground truth (mean 2.76, SD 1.90 per post). Moreover, they struggled to comprehend the context of the post content, often extracting medical jargon such as *side effect*, *lab report*, and *medication* as symptoms. Substantially, GHNLP and ACM extracted 64% (2874/4463) and 70% (5691/8161) incorrect symptoms, respectively, of the total symptoms identified (Figure 4). Longer posts aggravated these issues as the presence of more jargon further reduced the precision and *F*_1_-scores of these models (Table 3). However, both models achieved better recall than precision due to overprediction.

#### Baselines Struggle to Capture Nuanced Symptoms

All the baselines struggled to detect subtle symptoms, specifically when they were stated in informal language. As a result, GHNLP correctly identified only 50% (1589/3124) of the ground truth symptoms, whereas the LLMs showed better performance with approximately 65% (4133/6248) correct matches (Figure 4). Although ACM achieved a 79% (2470/3124) match with the ground truth, its overall performance was hindered by significant overpredictions. For instance, a PCOS symptom, *high testosterone*, was mentioned informally in the following post, but only LSE and none of the baselines could identify it:

I had two sets of blood tests taken last year, which showed that my hormones (especially testosterone) were all over the place. My GP said that my test results were enough to confirm PCOS diagnosis.

#### LSE Outperformed State-of-the-Art LLMs

Figure 4 shows that, while LLMs significantly reduced the number of incorrectly identified symptoms, they still missed a notable number of symptoms. Leveraging the symptom list generated by LSE in the base prompt for generative pretrained transformers increased the number of correctly identified symptoms, enhancing overall performance. The high values for LSE across all metrics in Table 3 indicate that the comprehensive symptom list effectively covered all PCOS lived experience symptoms while filtering out generic ones.

#### Statistical Significance

We also compared the *F*_1_-score of LSE with those of the baselines using the approximated randomization test [28] with a similarity threshold of 0.6 to determine whether the results were statistically significant. As this test does not require specific distributional assumptions, it is particularly suitable for complex metrics such as the *F*_1_-score [64]. We conducted pairwise comparisons between symptoms extracted from a post via LSE and each of the baselines. The number of occurrences in the sample set of 1000 posts in which LSE significantly outperformed the baselines at 95% (*P*<.05) and 90% (*P*<.10) confidence levels. The observed results suggest that, in terms of the *F*_1_-score, LSE significantly outperformed all the other baselines shown in Table S10 in Multimedia Appendix 1.

## Discussion

### Analysis of PCOS Symptomatology

#### Overview

Our study was motivated by the limitations of existing information extraction methods in identifying symptom expressions from colloquial patient narratives. We propose an LSE method incorporating LLMs into the pipeline to capture disease-specific symptoms from social media discourse. We focused on PCOS as a representative case given its highly variable and often underdocumented symptomatology. We evaluated LSE against multiple strong baselines across a manually annotated Reddit dataset, and it demonstrated substantial improvements in precision, recall, and *F*_1_-score.

We conducted an in-depth study of PCOS symptomatology to evaluate the characteristics, prevalence, and occurrence patterns of the symptoms, capturing implicit insights from patient-generated data. We also highlighted how capturing symptoms in patient-centric language can bridge gaps between patient experiences and clinical practice, potentially enhancing health care provider communication and guiding clinical decision-making.

#### Potentially Emerging Symptoms

**Figure 5:**
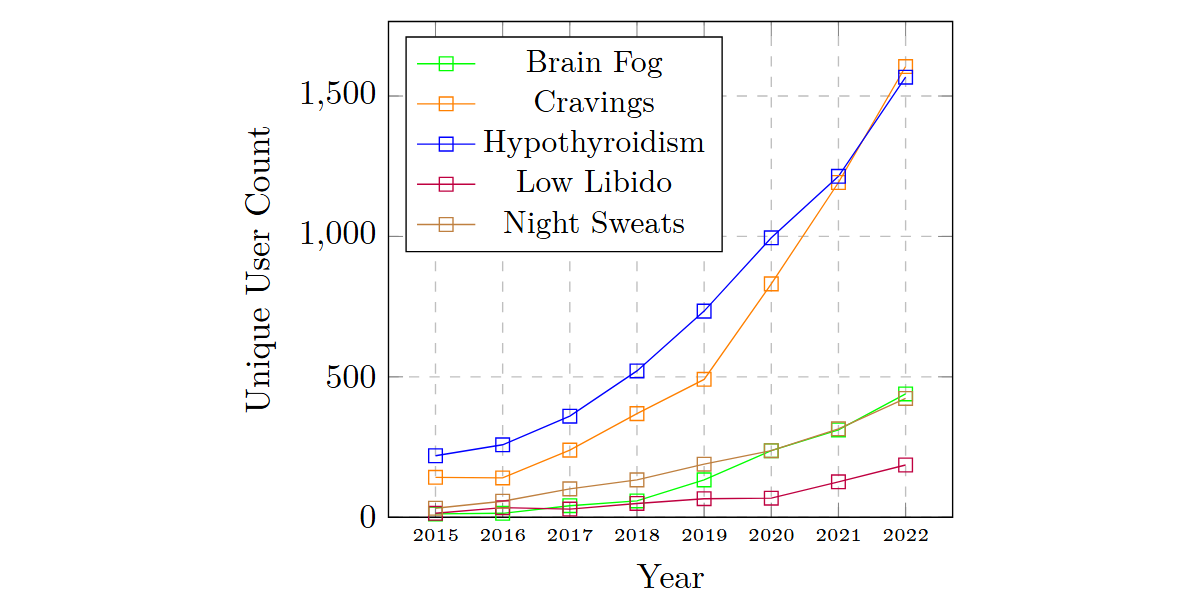
Instances of potentially emerging symptoms mentioned by users in the primary dataset, showing notable upward trends over time.

We identified 28 distinct symptoms from our comprehensive symptom list prominently reported by patients yet currently underrepresented or insufficiently explored in established medical literature and not acknowledged by existing eHealth sources (highlighted in italics in Table 2). Although many of these symptoms currently lack robust clinical evidence linking them directly to PCOS, their frequent occurrence in patient-generated data suggests that patients often experience and associate these symptoms with PCOS. As illustrated in Figure 5, the number of unique users reporting some of these symptoms, such as hypothyroidism and brain fog, demonstrate a consistent upward reporting trend over time. Moreover, some recent studies have provided medical evidence supporting the connection between some of these symptoms and PCOS, such as brain fog, thyroid disorder, low libido, digestive issues, and cravings [65-69]. Therefore, we marked them as potentially emerging symptoms. However, while frequently reported by patients, it is important to recognize that these symptoms might not represent entirely novel manifestations of PCOS. Instead, they may reflect less common but known clinical symptoms, secondary manifestations of the primary disease, or symptoms associated with frequently co-occurring conditions. For example, hypothyroidism, reported by 5366 users, reflects a well-established thyroid-PCOS comorbidity [66], underscoring potential shared pathophysiological mechanisms rather than indicating a novel PCOS symptom. In addition, nausea and cravings, although frequently reported by patients, advocate for a broader and patient-inclusive diagnostic perspective rather than confirming direct causality. These emerging findings reinforce the credibility of patient-reported underdocumented symptoms and underscore the necessity for rigorous clinical investigation to determine their prevalence, pathophysiological connections, and implications for diagnosis and treatment protocols.

#### Capturing Symptoms in Patient-Centric Language

LSE captured the lived experience symptoms as expressed by the patients. This approach makes the symptoms more accessible to laypersons compared to clinical terminology and jargon. Some symptoms with unorthodox clinical terms require complex medical tests to diagnose, whereas their effects are often evident in lived experiences, and hence, these symptoms are included in the symptom list. For instance, hyperandrogenism is a symptom that may not be familiar to all users and requires laboratory tests, whereas hirsutism, acne, or hair loss are visible outcomes of the condition that are easily recognized and commonly reported by users, as shown in Table 2.

#### Identifying Co-Occurrence of Symptoms

**Figure 6:**
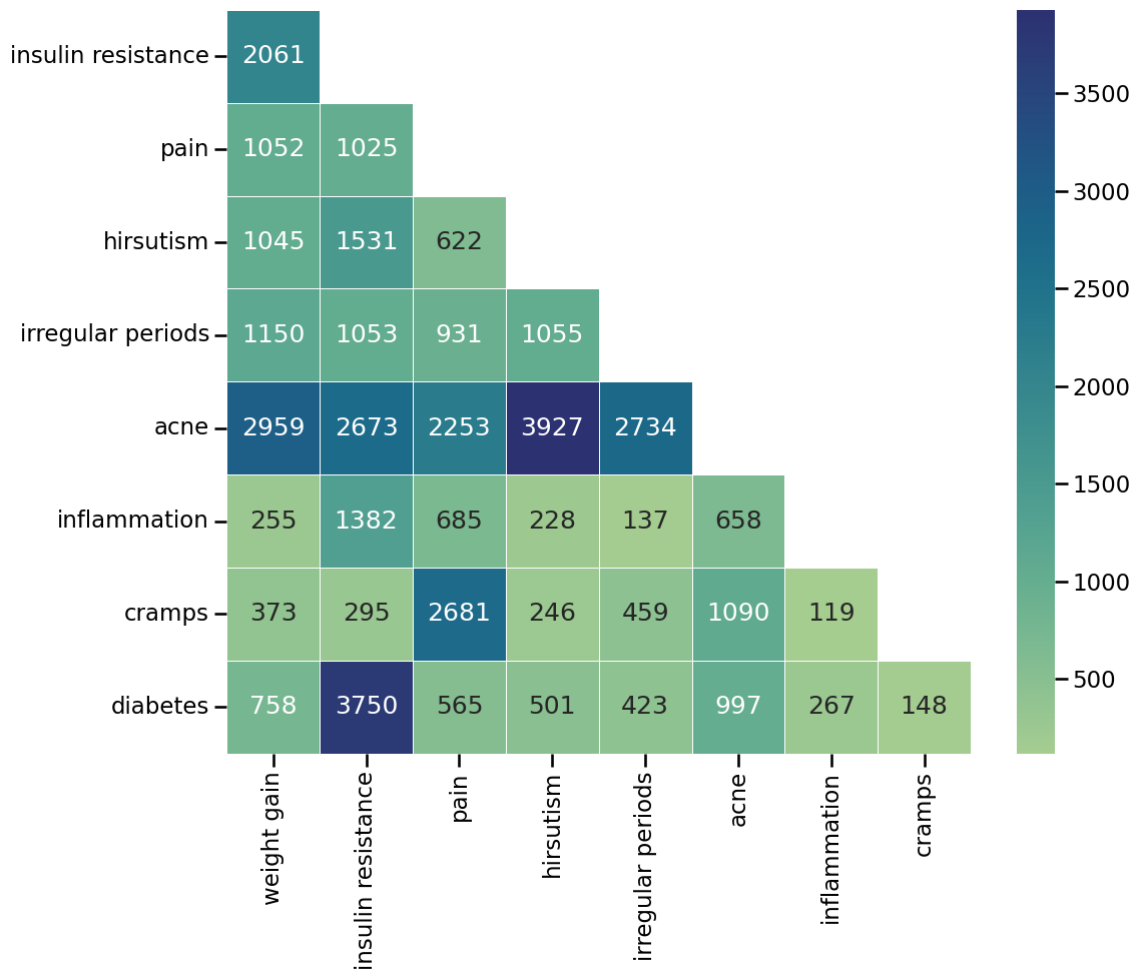
A segment of the symptom co-occurrence matrix, where hirsutism and acne appear as one of the most frequently mentioned symptom pairs.

We constructed a symptom co-occurrence matrix in which multiple symptoms mentioned by an individual in a single post or comment were considered as co-occurring. Figure 6 illustrates a segment of the matrix highlighting the most frequently mentioned symptom pairs with their number of co-occurrences. The eHealth forums further complemented these co-occurrences. Understanding these co-occurring symptoms raises awareness among the community and helps individuals better cope with their condition. In addition, such analysis can assist health care providers and patients in proactively diagnosing and managing symptoms such as hirsutism, oily skin, and acne, as observed in the following comment:

The best natural remedy I’ve tried is spearmint supplement. I’m a thin cysterbut have most of the other symptoms and it has been the only thing I’ve foundso far that helps with hirsutism, oily skin, acne.

#### Association With Chronic Diseases and Comorbidity

We identified 8 chronic diseases from the list of PCOS symptoms that were distinct medical conditions in their own right. For example, chronic conditions such as hypothyroidism and endometriosis were mentioned by 5366 and 2303 unique users, respectively (Table 2). The association with chronic diseases and comorbidity increases the complexity of the disease and its management and significantly impacts social determinants of health, as shown in the following example:

I went to the ER...but they found cysts on my ovaries and mentioned PCOS with possibly Endometriosis. Now, I have no insurance and I’m struggling to find the care. My husband and I want to start a family, but I feel like our chances are slipping away because I can’t afford the help I need. Any advice on where to get financial or medical support would be greatly appreciated.

The financial strain from frequent physician visits and prolonged medications impacts patients’ overall health outcomes. This emphasizes the need for clinical and public health research to find ways to reduce the burden on patients, such as overprescription and cost. This may also involve policy reforms to ensure comprehensive coverage for chronic conditions associated with PCOS.

#### Enhancing Patient–Health Care Provider Communication

The comprehensive symptom list generated by our lexicon-based approach, encompassing both widely recognized and emerging patient-reported symptoms, provides clinicians with a deeper insight into how patients perceive and articulate their condition. For example, terms such as *brain fog*, *pelvic pressure*, and *fatigue* are not only clinically relevant but also capture the nuanced ways in which patients describe their daily experiences with PCOS. Studies also show that using patient-generated health-related terminology during visits deepens shared understanding and decision‑making [70]. Therefore, clinicians can leverage this enriched vocabulary to enhance patient–health care provider communication, promoting empathy, validating patient experiences, and facilitating more targeted and personalized clinical interviews.

#### Guide to Clinical Decision-Making

The patient-derived symptom list (Table 2) can serve as a preliminary checklist during consultations to identify overlooked or underdiscussed symptoms that patients might hesitate to share spontaneously due to stigma or uncertainty. Documenting co-occurrence patterns further empowers clinicians to anticipate linked symptoms. A patient presenting with hirsutism, for instance, could be screened for insulin resistance or acne, enabling early intervention and holistic management. To scale these benefits, the symptom list can be integrated into electronic health record systems. This integration allows for auto-completion prompts that reflect patient language while supporting consistent clinical documentation. Such structured integration enhances clinical decision-making through automated reminders and promotes interoperability for population-level research. Acknowledging patient-reported symptoms does not undermine established clinical evidence; rather, it complements traditional clinical frameworks by incorporating patient perspectives into evidence-based medicine.

### Limitations

Despite the promising results, this study has several limitations. First, our study focused on a single health condition and a single social media platform, which may introduce platform-specific biases and limit generalizability. Second, both the lexicon and the evaluation sample sizes are relatively small for broader conclusions. Third, our findings are exploratory and hypothesis generating and not yet clinically validated.

We also acknowledge specific implementation limitations. Although the LSE pipeline is designed for modularity and adaptability, applying it to other complex health conditions may require empirical adjustments. Determining optimal clusters for symptom identification and establishing semantic mapping rules for symptom normalization would require condition-specific customization. In this study, we used silhouette scores and inertia analysis to determine the optimal cluster metrics, such as the number of clusters (*k*). However, we recognize the limitation of distance-based metrics due to the curse of dimensionality, which may not fully capture cluster shape or density nuances. Despite this, our bootstrap stability analysis demonstrated consistently high Adjusted Rand Index (ARI) values across various *k* settings, indicating that our symptom clusters were robust to moderate changes in the number of clusters and to the random removal of data points. The performance of this framework may vary depending on the extracted lexicon due to linguistic variability and the nature of symptom discourse. Future work should focus on validating this method across diverse conditions, platforms, and languages. In addition, we plan to conduct online surveys and focus groups involving both patients and practitioners to assess the clinical relevance of our findings.

### Conclusions

This work focused on extracting and analyzing disease-specific symptoms from individuals’ lived experiences with an application to the PCOS use case. The uniqueness of our work lies in several key aspects. The extraction of a symptom-related lexicon ensures the capture of subtle mentions without overlooking informal expressions on social media. We generated disease-specific symptoms by integrating LLMs into the NLP pipeline rather than relying on traditional NLP methods, which often obtain generic symptoms. In addition, analyzing PCOS symptomatology across varied dimensions revealed unique findings, such as emerging new symptoms expressed in patient-centric language, symptom co-occurrence patterns, and associated comorbidities. The outcomes of this work may help eliminate uncertainties surrounding complex medical conditions and contribute to clinical research and public health policies in a community-informed way.

## Appendix

Multimedia Appendix 1 Supplementary tables, prompts, and results.

## Abbreviations

ACM: Amazon Comprehend Medical
API: application programming interface
BERT: bidirectional encoder representations from transformers
DBCV: density-based clustering validation
GHNLP: Google Healthcare Natural Language Application Programming Interface
HDBSCAN: hierarchical density-based spatial clustering of applications with noise
JI: Jaccard index
LLM: large language model
LSE: lexicon-based symptom extraction
NLP: natural language processing
PCOS: polycystic ovary syndrome

## Data Availability

We have publicly released our curated Reddit dataset and human-annotated labeled dataset. To support reproducibility, all source code and resources used in this study have also been made available. The datasets and related materials can be accessed through our GitHub repository [71].
