# Supplementary material for: Extracting Symptoms of Complex Conditions From Online Discourse (Subreddit to Symptomatology): Lexicon-Based Approach
Source: JMIR Med Inform. 2025 Sep 12;13:e70940. doi: 10.2196/70940 (PMC12475878; doi:10.2196/70940)
Supplement: Multimedia Appendix 1 [file medinform_v13i1e70940_app1.doc]

# **Appendix**

**Table S1.** Summary of steps to describe the criteria and rule-based methods used to remove irrelevant texts from social media contents.

| 1. We remove the posts or comments authored by "[deleted]" users and whose content is either "[deleted]" or "[removed]" as these entries contain no meaningful text. 2. We remove posts containing fewer than five words as it is difficult to obtain meaningful insights from fewer words. 3. We remove URL-only or link-dominant posts using a two part filtering step:    1. **Personal experience keywords** – We check for words like *“I,” “me,” “my,” “myself,” “I’ve,” “I’m,”* and *“I’d.”*    2. **URL-to-text ratio** – We measure the proportion of the post’s text occupied by URLs.   **Removal Criteria:**   1. Posts with fewer than two personal keywords 2. Posts where URLs made up more than 40% of the text 3. We set the 40% threshold after analyzing posts under the *"General/Advice"* flair, since we observe that these posts are generally suggestions, references, or promotions, and lack personal health experiences . |
| --- |

**Figure S1.** The distribution of frequent uni-grams in General/Advice and Meds/Supplement Flair.


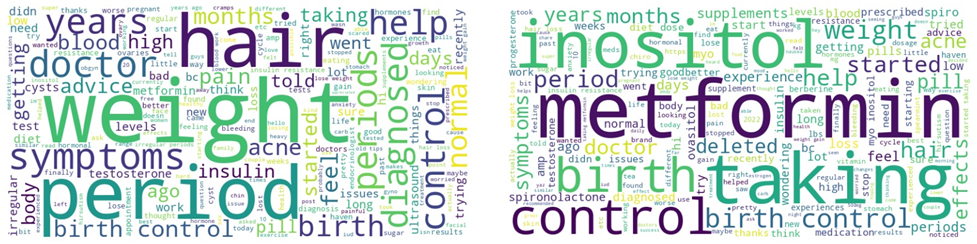


(a) General/Advice Flair

**Table S2.** Summary on different flairs and distribution of associated posts in r/PCOS subreddit after data preprocessing.

| Flair Name | Posts (count) |
| --- | --- |
| General/Advice Genera | 14180 |
| Meds/Supplements | 7218 |
| Period | 3742 |
| Rant/Venting | 2933 |
| General Health | 2776 |
| Weight | 2032 |
| Hair Loss/Thinning | 1872 |
| Hirsutism | 1631 |
| Fertility | 1512 |

**Figure S2.** Illustrates the (zero-shot) prompt for extracting symptom-related keyphrases.

##
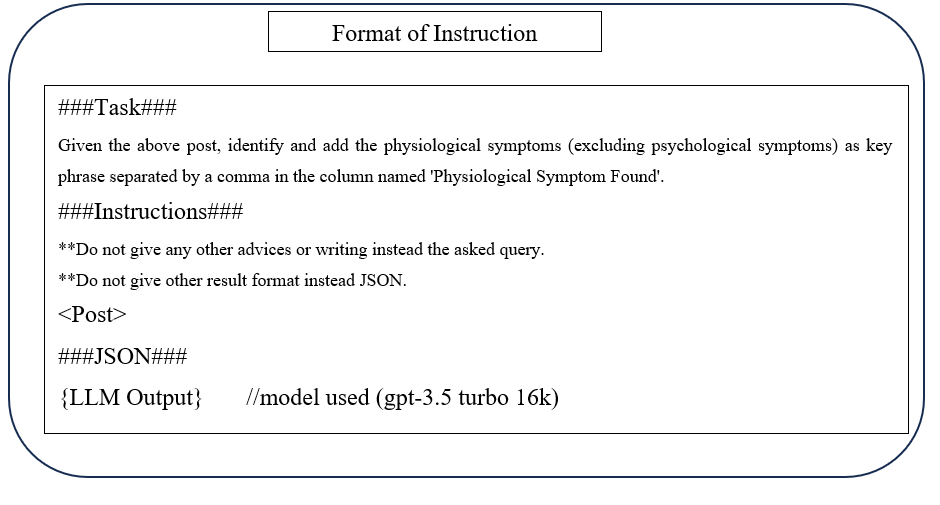


## **Table S3.** Number of unique symptom related keyphrases added to the human annotated dataset shows diminishing returns.

| Number of Posts | Noumber of unique new keyphrases added |
| --- | --- |
| 0-200 | 148 |
| 201-300 | 33 |
| 301-400 | 25 |
| 401-500 | 21 |

## **Table S4.** The distribution of annotation errors in the Symptom-related lexicon extraction of LSE are categorized in the below classes.

| Class | | Definition | Example | Error% |
| --- | --- | --- | --- | --- |
| C1 | Partial/Omitted Phrase | Only part of a multi-word symptom expression is extracted or key components are omitted, | *“Hair thinning on scalp”* extracted as just *“hair”* | 26% |
| C2 | Ambiguously Expressed | Instances where symptoms are described using vague or colloquial language, making interpretation uncertain. In such cases, either the LLM or the human annotator may choose to extract the symptom, while the other may ignore it | *“Does anyone also not have insulin resistance, I was tested with a two hour glucose test and nothing it was normal.”* The LLM extracted *“no insuline resistance”* as a symptom keypharse, while the human annotator ignored it as the test result was normal. | 24% |
| C3 | Implicit Mention | The symptom is implied through descriptive language rather than explicitly stated, making it harder for automated systems to detect | *“haven’t bled in 6 months”* implying *missed periods* | 23% |
| C4 | Hallucination /Other | GPT hallucinates by ignoring symptoms not known as PCOS symptoms or by generating its known PCOS symptoms, | 1. Patient mentions *“joint pain”* (non-classic PCOS symptom) → LLM ignores it.  2. LLM invents *“ovarian cysts”* even if unmentioned in the text. | 20% |
| C5 | Entity type confusion | Cases where non-symptom entities such as medication names, clinical tests, or anatomical terms are extracted as symptoms due to their surface form or contextual ambiguity | *`Metformin’*, `insulin level’, `endocrinologist’ are incorrectly identified as a symptom | 7% |

## **Table S5.** Summary of quantitative analysis on HDBSCAN (in different settings)

| Method | Number of clusters | DBCV score | Noise % |
| --- | --- | --- | --- |
| HDBSCAN (mcs = 100, ms = 50) | 50 | 0.33 | 54% |
| HDBSCAN (mcs = 65, ms = 65) | 65 | 0.43 | 56% |
| HDBSCAN optimal (mcs = 50, ms = 50) | 94 | 0.49 | 52% |
| HDBSCAN(mcs = 10, ms = 20) | 279 | 0.63 | 39% |
| HDBSCAN (mcs = 10, ms = 5) | 621 | 0.64 | 31% |

We ran a grid of HDBSCAN in below settings:

- Min_cluster_size (mcs) ∈ {10, 15, 20, 25, 30, 35….100
- min_samples      ∈ {mcs/2, mcs}

For each run we computed:

- DBCV (cosine): measures how dense and well-separated core clusters are
- Noise fraction (points with label = −1)
- Symptom‑coverage loss: % of symptoms from comprehensive symptom list (64) that method could not identify but fell into the noise bin

We compared different HDBSCAN settings (mcs, ms) using various metrics such as Cluster number, DBCV score, Noise%, and Symptom coverage % , where DBCV scores are only computed over core (clustered) points, excluding noise in HDBSCAN.

**Table S6.** Summary on sensitivity and stability analysis of choice ‘k’

| Method | Number of clusters | Silhouette score | Median ARI | 95% CI |
| --- | --- | --- | --- | --- |
| K-means (k=106) | 106 | 0.33 | 0.78 | [0.75, 0.82] |
| K-means (k=96) | 96 | 0.32 | 0.76 | [0.73, 0.80] |
| K-means (k=86) | 86 | 0.31 | 0.75 | [0.72, 0.79] |

We evaluate a bootstrap stability test at k = 86, 96, and 106 as follows. For each value of k, we performed on 500 random subsamples consisting of 80 % of the data without replacement. Each subsample was reclustered with k-means (same initialization and parameters as the full‐data model), and the resulting labels were compared to the full‐data labels using the Adjusted Rand Index (ARI). We report the results of Median ARI and 95% CI in the below table.

## **Table S7. Ruleset used for Semantic Mapping in Symptom Normalization Step.**

| Step | Rule | Example |
| --- | --- | --- |
| R1: Synonym Merging | Clusters with semantically identical or highly overlapping terms were grouped under a primary symptom. | Cluster 1 ("irregular periods") merged with Cluster 11 ("irregular bleeding"), Cluster 14 ("irregular menstrual cycles"), and Cluster 91 ("irregular cycles") into a single symptom: "Irregular periods". |
| R2: Informal to Standard term mapping | Patient-centric expressions (cluster) were mapped to primary symptom (standardized clinical terms) for consistency when pathophysiology is identical. | Cluster 5 ("Fatigue"), Cluster 58 ("tiredness") Cluster 62 ("weakness"), and Cluster 83( "low energy") were combined into "Fatigue. |
| R3: Anatomical Redundancy | Merge phrases that denote the same organ/location without adding new clinical meaning. | Cluster 29 (“enlarged ovaries”), Cluster 56 (“Cysts”) and Cluster 93 “follicles*”)*  mapped to Cluster 7 “cysts on ovaries”. |
| R4: Distinct pathophysiology preserved | Do not merge if two terms represent clinically different phenomena. | "Pelvic pain" (Cluster 51) was retained as distinct from "abdominal pain" (Cluster 75) based on anatomical specificity. |

**Table S8.** Summary of Data distribution in terms of word length in the posts and comments.

|  | Number of samples | Number of avg word/sample | word_length<=100  (%) | 100<word_length<=300  (%) | 300<word_  Length  (%) |
| --- | --- | --- | --- | --- | --- |
| Post | 59,962​ | 152.14 | 44.58 | 44.85 | 10.0 |
| Comment | 641,441​ | 59.57 | 83.41 | 14.8 | 1.45 |

## **Table S9.** The comparison on coverage of our comprehensive PCOS symptom list (LSE) with e-health forums are provided in detail.

| Comprehensive Symptom List (LSE) | Mayo Clinic | WebMD | ICD-10 | Johns Hopkins Medicine | NHS | CDC | WHO | Other | Emerging symptoms |
| --- | --- | --- | --- | --- | --- | --- | --- | --- | --- |
| Hirsutism | **✓** | **✓** | **✓** | **✓** | **✓** | **✓** | **✓** | X | X |
| Acne | **✓** | **✓** | **✓** | **✓** | **✓** | **✓** | **✓** | X | X |
| Cysts on Ovaries | **✓** | **✓** | **✓** | **✓** | **✓** | X | **✓** | X | X |
| Pain | X | **✓** | X | X | X | X | X | X | X |
| Weight Gain | **✓** | **✓** | **✓** | **✓** | **✓** | **✓** | **✓** | X | X |
| Mood Swings | X | **✓** | X | X | **✓** | **X** | X | X | X |
| Insulin Resistance | **✓** | **✓** | **✓** | **✓** | **✓** | **X** | X | X | X |
| Irregular Periods | **✓** | **✓** | **✓** | **✓** | **✓** | **✓** | **✓** | X | X |
| Infertility | **✓** | **✓** | **✓** | **✓** | **✓** | **✓** | **✓** | X | X |
| Diabetes | **✓** | **✓** | **✓** | **✓** | **✓** | **✓** | X | X | X |
| Cramps | X | **✓** | X | X | X | X | X | X | X |
| Spotting | X | X | X | **✓** | X | X | X | X | X |
| Hypothyroidism | X | X | X | X | X | X | X | X | **✓** |
| No Period | X | **✓** | X | **✓** | **✓** | X | **✓** | X | X |
| Anovulation | **✓** | **✓** | **✓** | **✓** | **✓** | X | **✓** | X | X |
| Nausea | X | X | X | X | X | X | X | X | **✓** |
| Fatigue | X | **✓** | X | X | X | X | X | X | X |
| Cravings | X | X | X | X | X | X | X | X | **✓** |
| Bloating | X | X | X | **✓** | X | X | X | X | X |
| Headaches | X | **✓** | X | X | X | X | X | X | X |
| Hair Loss | **✓** | **✓** | **✓** | **✓** | **✓** | **✓** | **✓** | X | X |
| High Testosterone | **✓** | **✓** | **✓** | **✓** | **✓** | X | **✓** | X | X |
| Heavy Periods | X | **✓** | X | X | X | X | X | X | X |
| Skin Issues | **✓** | **✓** | **✓** | **✓** | **✓** | **✓** | X | X | X |
| Hormonal Imbalance | **✓** | **✓** | **✓** | **✓** | **✓** | X | **✓** | X | X |
| Inflammation | **✓** | **✓** | X | X | X | X | X | X | X |
| Endometriosis | X | X | X | X | X | X | X | X | **✓** |
| Painful Periods | X | X | X | X | X | X | X | X | **✓** |
| Insomnia | X | **✓** | X | X | **✓** | **✓** | X | X | X |
| Loss of Appetite | X | X | X | X | X | X | X | X | **✓** |
| High DHEAS | X | X | X | X | X | X | X | X | **✓** |
| Dizziness | X | X | X | X | X | X | X | X | **✓** |
| Night Sweats | X | X | X | X | X | X | X | X | **✓** |
| Blood Clots | X | X | X | X | X | X | X | X | **✓** |
| Stomach Pain | X | X | X | X | X | X | X | X | **✓** |
| Anemia | X | X | X | X | X | X | X | X | **✓** |
| Brain Fog | X | X | X | X | X | X | X | X | **✓** |
| Vomiting | X | X | X | X | X | X | X | X | **✓** |
| High Androgen Levels | **✓** | **✓** | **✓** | **✓** | **✓** | X | **✓** | X | X |
| Oily Skin | X | **✓** | **✓** | **✓** | X | **✓** | **✓** | X | X |
| Back Pain | X | X | X | X | X | X | X | X | **✓** |
| Constipation | X | X | X | X | X | X | X | X | **✓** |
| Pelvic Pain | X | **✓** | **✓** | **X** | X | **✓** | X | X | X |
| Digestive Issues | X | X | X | X | X | X | X | X | **✓** |
| Belly Fat | X | **✓** | X | **✓** | X | X | **✓** | X | X |
| Acanthosis Nigricans | X | **✓** | **✓** | **✓** | X | **✓** | X | X | X |
| High Cholesterol | X | X | **✓** | X | **✓** | **✓** | X | X | X |
| High Insulin Levels | X | **✓** | X | X | **✓** | X | **✓** | X | X |
| Low Libido | X | X | X | X | X | X | X | X | **✓** |
| Abdominal Pain | X | X | X | X | X | X | X | X | **✓** |
| Dry Skin | X | X | X | X | X | X | X | X | **✓** |
| Ovary Pain | X | X | X | X | X | X | X | X | **✓** |
| High Estrogen | X | **✓** | X | X | X | X | X | X | X |
| Low Estrogen | X | X | X | X | X | X | X | **✓** | X |
| Stretch Marks | X | X | X | X | X | X | X | X | **✓** |
| Brown Discharge | X | X | X | X | X | X | X | **✓** | X |
| Skin Tags | X | **✓** | X | **✓** | X | X | X | X | X |
| Breast Tenderness | X | X | X | X | X | X | X | X | **✓** |
| High Prolactin | X | X | X | X | X | X | X | X | **✓** |
| Pain During Sex | X | X | X | X | X | X | X | X | **✓** |
| Frequent Urination | X | X | X | X | X | X | X | X | **✓** |
| Weight Loss | X | X | X | X | X | X | X | X | **✓** |
| Redness | X | X | X | X | X | X | X | X | **✓** |
| High LH Levels | X | X | X | X | X | X | **✓** | **✓** | X |

**Figure S3.** Illustrates the (zero-shot) prompt for extracting symptoms using Baseline LLMs.**
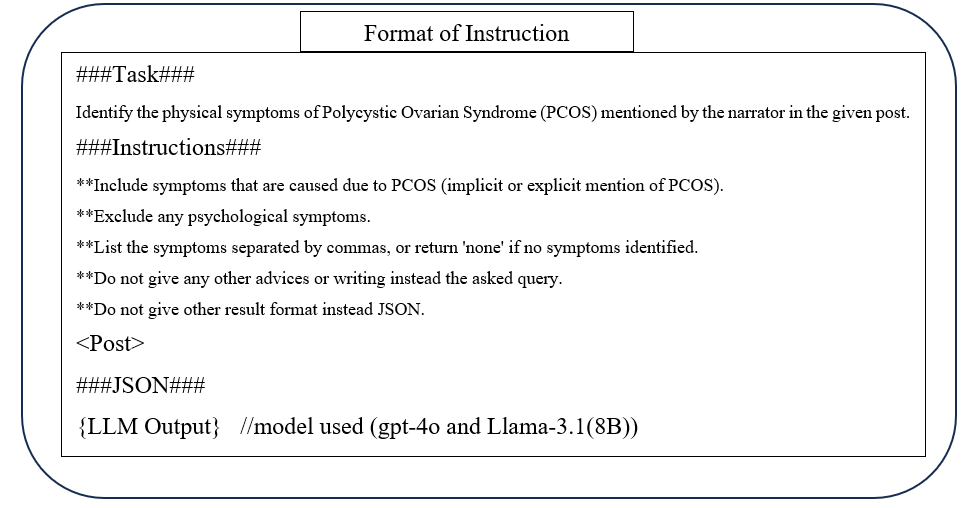
**

**Figure S4.** Illustrates the LSE-guided prompt for extracting symptoms.

**
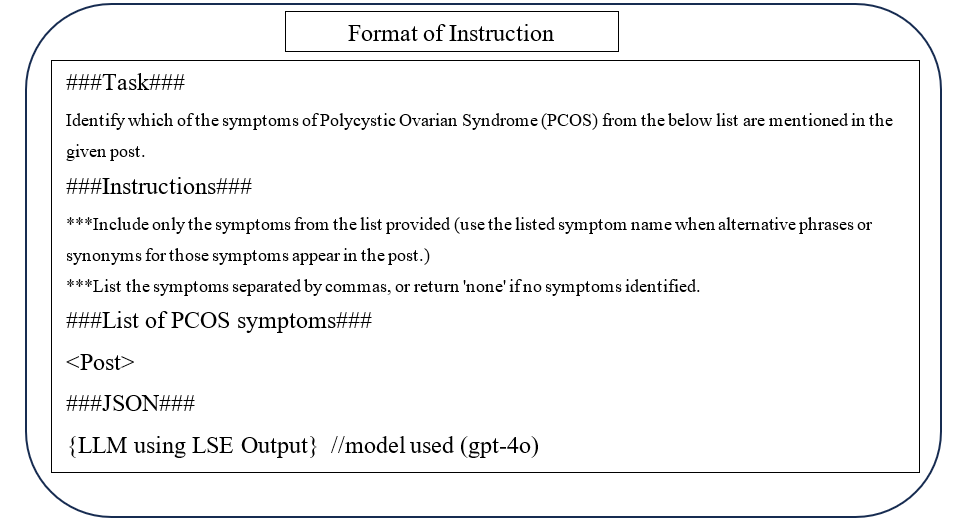
**

##

**Table S10.** Number of occurrences in a sample of 1000 posts in which lexicon-based symptom extraction demonstrated statistically significant results in terms of the *F*_1_-score compared to the baselines (Amazon Comprehend Medical [ACM], Google Healthcare Natural Language Application Programming Interface [GHNLP], Llama, and GPT-4) at *P*<.05 and *P*<.10.

|  | ACM, number of posts | GHNLP, number of posts | Llama, number of posts | GPT-4, number of posts |
| --- | --- | --- | --- | --- |
| *P*<.05 | 735 | 745 | 605 | 675 |
| *P*<.10 | 780 | 795 | 665 | 710 |

##

## 
